# Antioxidant Capacity, Phytochemicals, Minerals, and Chemical Pollutants in Worker Honey Bee (*Apis mellifera* L.) Broods from Northern Thailand: A Safe and Sustainable Food Source

**DOI:** 10.3390/foods13131998

**Published:** 2024-06-25

**Authors:** Phannika Tongchai, Sumed Yadoung, Kunrunya Sutan, Saweang Kawichai, Khanchai Danmek, Jakkrawut Maitip, Sampat Ghosh, Chuleui Jung, Bajaree Chuttong, Surat Hongsibsong

**Affiliations:** 1 School of Health Sciences Research, Research Institute for Health Sciences, Chiang Mai University, Chiang Mai 50200, Thailand; phannika_tongchai@cmu.ac.th; 2 Environmental Science Program, Faculty of Sciences, Chiang Mai University, Chiang Mai 50200, Thailand; sumed_y@cmu.ac.th; 3 Environmental, Occupational Health Sciences and NCD Center of Excellence, Research Institute for Health Sciences, Chiang Mai University, Chiang Mai 50200, Thailand; kunrunya.s@cmu.ac.th (K.S.); sawaeng.k@gmail.com (S.K.); 4 School of Agriculture and Natural Resources, University of Phayao, Phayao 56000, Thailand; khanchai.da@up.ac.th; 5 Faculty of Science, Energy and Environment, King Mongkut’s University of Technology North Bangkok, Rayong Campus, Bankhai, Rayong 21120, Thailand; jakkrawut.m@sciee.kmutnb.ac.th; 6 Agriculture Science and Technology Research Institute, Andong National University, Andong 36729, Republic of Korea; sampatghosh.bee@gmail.com (S.G.); cjung@andong.ac.kr (C.J.); 7 Department of Plant Medicals, Andong National University, Andong 36729, Republic of Korea; 8 Meliponini and Apini Research Laboratory, Department of Entomology and Plant Pathology, Faculty of Agriculture, Chiang Mai University, Chiang Mai 50200, Thailand

**Keywords:** the antioxidant activity, phytochemical, healthy food, honey bee brood, nutritional security, safety, heavy metal

## Abstract

Honey bee brood (HBB) (*Apis mellifera* L.), a traditional protein source, has been studied for its nutritional value, but bio-functional properties and safety concerns have not been verified. This study examined the Antioxidant capacity, phytochemicals, minerals, and chemical pollutants in worker broods from several apiaries in Northern Thailand. HBB samples were lyophilized to evaluate antioxidant capacity using ABTS, DPPH, and FRAP assays, tests with water, and 70% ethanol extracts. Phytochemicals were identified using LC-QTOF-MS; pollutants were analyzed chromatographically, and minerals were determined using ICP-OES. The results showed that the evaluated antioxidant capacity of the ethanol extracts included DPPH 2.04–3.37 mg/mL, ABTS 21.22–33.91 mg/mL, and FRAP 50.07–104.15 mg AAE/100 g dry weight. Water extracts had outstanding antioxidant activities except for ABTS, with DPPH 10.67–84.97 mg/mL, ABTS 9.25–13.54 mg/mL, and FRAP 57.66–177.32 mgAAE/100 g dry weight. Total phenolics and flavonoids in ethanol extracts ranged from 488.95–508.87 GAE/100 g to 4.7–12.98 mg QE/g dry weight, respectively. Thirteen phytochemicals were detected and contained adequate mineral contents in the HBBs from different locations found, which were K, Ca, Mg, and Na, and no heavy metals or pollutants exceeded safe levels. These results imply that HBB from different apiaries in Northern Thailand is a nutritious food source with considerable antioxidants and a safe and sustainable food source.

## 1. Introduction

Honey bees (*Apis mellifera* L.), commonly known as Western honey bees, are indeed widely recognized and extensively utilized in beekeeping and honey production [1]. Since time immemorial, human civilization has shared a profound and enduring connection with honey bees, treasuring their precious gift of honey not only as a delectable food source but also as a medicine, a testament to the enduring harmony between mankind and these remarkable creatures [2]. Until recent times, the focus of our interaction with honey bees has predominantly centered on the extraction of various hive products, including honey, wax, propolis, royal jelly, and bee venom [3]. Surprisingly, the bees themselves, although consumed by people in Asia and Africa in various instances throughout history, have received far less recognition [1,4,5,6,7,8,9]. Nevertheless, in recent years, there has been a growing interest in honey bees as a potential food and feed source [10]. The scientific literature now exists that explores their nutritional value, shedding light on the remarkable benefits they offer [11].

Honey bee brood (HBB) plays a pivotal role in global ecosystems by serving as pollinators for a diverse array of crops and wild plants [3]. The health and well-being of honey bee colonies are critical not only for honey production but also for maintaining biodiversity and ensuring food security [12,13,14,15]. Understanding the nutritional composition of HBB is essential, as it directly influences the overall vitality and resilience of the colony [16]. Honey bee brood’s nutritional composition is of interest for human consumption for several reasons. Notably, it has been shown that HBBs have a high nutritional value. They have the greatest protein content, at 50.4%, followed by carbs at 22.1%, and fat at 20.5% [17,18]. Furthermore, essential minerals and vitamins abound in insects, with varying amounts contingent upon the species and age of the bug compared to adults. The larval stage—such as silkworms, which are said to be another protein-rich meal that has a high mineral content and a very high vitamin A content—is 273.99 mg/100 g of dry weight, which offers 400–500 kcal of energy per 100 g of dry weight [17,18].

The antioxidant capacity in HBB is a crucial aspect influenced by various factors. Studies have shown that honey bee products like drone brood homogenate exhibit significant antioxidant properties, with freeze-dried and sterilized HBB showing increased radical-scavenging activity and total reducing capacity [19,20]. Antioxidant capacity, a key parameter in assessing cellular defense mechanisms, is crucial for protecting HBB from oxidative stress [21]. Phytochemicals derived from the diverse floral sources that bees forage on not only provide essential nutrients but may also confer additional health benefits [22]. Furthermore, the antioxidant protection in honey bees varies with age, with older bees displaying higher total antioxidant capacity (TAC) and being less susceptible to pesticide toxicity, highlighting the importance of age-related antioxidant defenses in honey bees [23]. HBB is rich in minerals such as potassium, magnesium, calcium, and phosphorus, making them a valuable source of essential nutrients [11,17,18]. The mineral content of HBB is vital for proper growth, development, and metabolic functions, while the presence of chemical pollutants raises concerns about potential threats to bee health and, consequently, the ecosystems they support [24]. However, there has been relatively limited effort to explore the bio-functional compounds within HBBs. It is well-known that honey bees frequently forage in agricultural areas, heightening the risk of chemical pollutant accumulation, such as pesticides, within their hives [25]. This could result in contamination of adult bees, beeswax, and broods, thereby posing potential health risks for consumers of bee products.

The local population in Thailand acknowledges bee brood consumption, but it remains a somewhat niche aspect of their culinary tradition rather than being part of the mainstream food culture. There is a pressing need to investigate the bioactive components, mineral content, and chemical contaminants present in HBBs. While recent efforts have been made to investigate the nutritional potential of honey bees, issues such as bio-functional properties and safety concerns remain unresolved. This study investigated the antioxidant capacity, phytochemicals, minerals, heavy metal levels, and chemical contaminants to enhance understanding of the quality and safety issues associated with honey bee worker broods in Northern Thailand.

## 2. Materials and Methods

### 2.1. HBB Samples

**Figure 1:**
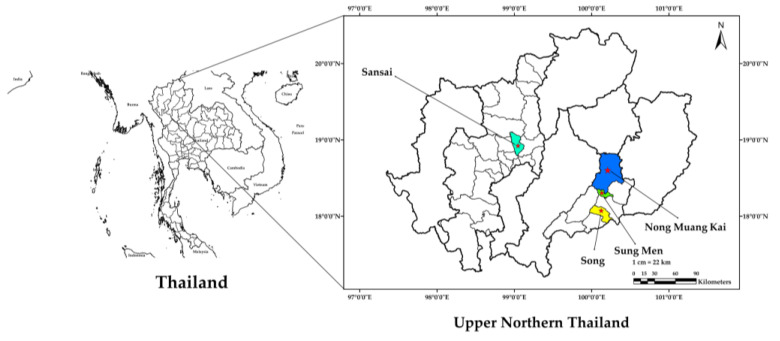
Geographical positioning of the HBB collection site in the northern region of Thailand.

Five samples of HBB were collected from apiaries or honey bee farms in Northern Thailand, including Nong Muang Kai District, Song District, Sung Men District Phrae Province, and San Sai District Chiang Mai Province (Figure 1) in November 2022. The samples were separated from honeycombs by being passed under hot steam. This method ensured that the brood was gently and effectively removed without damaging the samples. Once separated, the samples were immediately frozen at −20 °C until further analysis. Prior to analysis, these samples were freeze-dried to complete dryness using the SP VirTis Genesis Pilot Freeze Dryer (SP Stone Ridge, NY 12484, USA). The brood consisted primarily of pupae, with larvae making up less than 10% of the total. The resulting dried samples were ground into a powder and stored in a −20 °C freezer for further analysis. The water content of the bee brood samples fell within the range of 68.75–77.51% (Table 1).

### 2.2. Bioactive Compounds Analysis

#### 2.2.1. Extraction of HBB

One g of lyophilized HBB samples was subjected to an extraction process using 10 mL of deionized water and 70% ethanol. The samples were shaken at 2500 rpm for 5 min at room temperature. Subsequently, the samples were filtered using Whatman No. 1 filter paper and stored in a refrigerator at 4 °C before further analysis.

#### 2.2.2. DPPH Radical-Scavenging Activity

The DPPH radical-scavenging activity of the HBB extract was conducted following the method described by Siok Peng Kek et al. [26]. This study employed a 1,1-diphenyl-2-picryhydrazyl (DPPH) assay for analysis. A total of 100 µL of HBB extract solution was mixed with 100 µL of DPPH working solution in the 96-well plate. Subsequently, the mixture was incubated in the dark for 30 min. The absorbance at 517 nm was then determined using a microtiter plate reader (SPECTRO Star Nano) to calculate the percentage of DPPH-free radical-scavenging activity, and DPPH was determined by applying a calibration curve range of 1.562–100 μg/mL. The DPPH free radical-scavenging activity was calculated using the formula DPPH = [A_control_ − A_sample_]/A_control_ × 100, where A_control_ and A_sample_ were absorbance readings. The IC50 value, indicating the sample concentration needed to inhibit 50% of DPPH free radicals, was determined from the curve relating DPPH scavenging activity percentages to sample concentration.

#### 2.2.3. ABTS Radical-Scavenging Activity

The ABTS radical-scavenging activity was carried out following the experimental procedure outlined by Arnao et al. [26]. A stable radical, ABTS reagent, was employed in an aqueous solution containing 2,2-casino-bis (3-ethylbenzothiazoline-6-sulfonic acid) diammonium salt, with Trolox serving as a reference. To a 96-well plate containing 190 µL of ABTS working solution, 10 µL of HBB extract solution was added. After incubating in the absence of light for 10 min, absorbance was measured at a wavelength of 734 nm, and a calibration curve range of 3.125–100 μg/mL was used to determine ABTS. The ABTS radical-scavenging effect was calculated using the following formula: ABTS radical-scavenging effect % = ((Ab − Aa)/Ab) × 100, where Ab represents the absorbance of the ABTS radical with methanol, and Aa is the absorbance of ABTS radicals in the sample extract.

#### 2.2.4. Ferric Ion Reducing Antioxidant Power Assay (FRAP)

The FRAP test was performed following the experimental procedure outlined by Kek et al. [27]. The FRAP reagent, a solution, was formulated by mixing 2.5 mL of a 10 mM TPTZ (2,4,6-tripyridyl-s-trizine) solution in 40 mM HCl, 2.5 mL of a 20 mM ferric chloride (FeCl_3_) solution, and 25 mL of a 300 mM acetate buffer (pH of 3.6). Ten microliters (10 µL) of the diluted HBB extract solution was added with 190 µL of FRAP reagent in a 96-well plate. After 30 min in the absence of light, the sample’s absorbance was measured at a wavelength of 593 nm using a microtiter plate reader, and FRAP was determined by applying a calibration curve range of 1.562–100 μg/mL. The quantification of the outcome was expressed as milligrams of ascorbic acid equivalent per 100 g of HBB extract (mg AAE/100 g).

#### 2.2.5. Total Phenolic Content (TCP)

The total phenolic content was determined using the Folin–Ciocalteu method, adapted with minor modifications from the procedure outlined by Dewanto et al. [28]. Gallic acid (Fluka, 9470 Buchs, Switzerland) served as the standard substance in this methodology. Thirteen microliters (13 µL) of the HBB extract solution sample was combined with 13 µL of the Folin–Ciocalteau solution (MERCK, 82024, Taufkirchen, Germany), diluted with 10 µL of distilled water. After a 6-min interval, a solution consisting of 100 μL of distilled water and 125 μL of 7% Na_2_CO_3_ was added. Subsequently, the mixture underwent a 90-min incubation at room temperature. Absorbance was subsequently determined using a microplate reader (SPECTRO Star Nano) at a wavelength of 760 nm. The phenolic concentration was determined by applying a calibration curve range of 20–1000 μg/mL, and the results were expressed in gallic acid equivalents per gram of sample (mg GAE/100 g sample).

#### 2.2.6. Total Flavonoid Content (TFC)

The determination of the total flavonoid content followed the method outlined by Miliauskas et al. [29]. Twenty-five microliters (25 μL) of HBB extraction solution was mixed with 8 μL of 7% NaNO_2_ and 12.5 μL of distilled water and vigorously stirred. The solution was allowed to stand at room temperature for five minutes. Subsequently, 15 µL of a 10% AlCl_3_ solution was added, and the components were left at room temperature for another five minutes. Further, 50 μL of 1M NaOH solution and 28 µL of distilled water were added. After thorough mixing, the mixture was kept at room temperature for an additional five minutes. Absorbance was measured at 510 nm using a SPECTRO Star Nano microplate reader. The quantification of flavonoids was accomplished through the utilization of calibration curves range 10–500 μg/mL. The results were measured and expressed as quercetin equivalents per gram of sample (mg QE/g sample).

### 2.3. LC-Q-TOF/MS Identification of Phytochemicals

Phytochemical analysis was conducted using an ultra-performance liquid chromatography system coupled with a quadrupole time-of-flight mass spectrometer (Q-TOF-MS). Agilent 1290 Infinity II series coupled to the 6546 instruments (Agilent Tech., Santa Clara, CA, USA) in both positive and negative ionization modes, as previously described in standard methodologies [30,31]. The quantification of phytochemicals was performed through the utilization of the Medlin Library.

### 2.4. Mineral Analysis

#### 2.4.1. Sample Preparation

The dried HBB samples, weighing 0.2 g, were placed in a 100 mL Teflon vessel. The microwave extraction technique was employed for sample extraction. Nine milliliters of nitric acid and 1.0 mL of hydrogen peroxide were added to the samples, which were then placed in a 210 °C oven at 18,000 watts. The microwave extraction process lasted for 35 min, and the sample was allowed to cool to room temperature. The resulting mixture was combined with 1 mL of distilled water, vortex-mixed, and filtrated using a 0.2-µm syringe filter prior to analysis in inductive-coupled plasma optical emission spectrometry (ICP-OES, Agilent 5800 series, Stevens Creek Blvd., Santa Clara, CA, USA).

#### 2.4.2. ICP-OES Determination of Minerals

The mineral composition of HBB samples was determined according to the study of Kawichai et al. [32] with certain modifications, which are detailed as follows.

The analysis of minerals was conducted utilizing ICP-OES. The minerals that were investigated in this study included Ag (328.068 nm), As (188.980 nm), B (249.772 nm), Ba (455.403 nm), Be (313.042 nm), Ca (422.673 nm), Cd (214.439 nm), Co (238.892 nm), Cr (267.716 nm), Cu (327.395 nm), Fe (238.204 nm), K (766.491 nm), Mg (279.553 nm), Mn (257.610 nm), Mo (202.032 nm), Na (589.592 nm), Ni (231.604 nm), Pb (220.353 nm), Sb (206.834 nm), Se (196.026 nm), Si (251.611 nm), Ti (336.122 nm), Tl (190.794 nm), V (292.401 nm), and Zn (213.857 nm). ICP-OES condition: Argon 99.995% was used for analysis and axial detection; the standard concentration of all minerals was calibration curves range 0.01–10 μg/mL; and 1% nitric acid was used as a reagent blank.

### 2.5. Chemical Pollutants Analysis

#### 2.5.1. Organophosphate and Synthetic Pyrethroid Insecticide Analysis

The process of extracting HBB samples involved techniques adapted from previous studies with slight modifications [33]. Standard stock solutions of organophosphates (OPs) and pyrethroids (Pys) were employed. The OPs included methamidophos, mevinphos, diazinon, dicrotophos, monocrotophos, dimethoate, pirimiphos-methyl, chlorpyrifos, parathion-methyl, malathion, fenitrothion, prothiophos, methidathion, profenphos, ethion, triazophos, EPN, and azinphos-ethyl, while the Pys encompassed enpropathrin, lambda-cyhalothrin, permethrin, cyfluthrin, cypermethrin, fenvalerate, esfenvalerate, and dethamethrin. These standards were thoroughly prepared in ethyl acetate and methanol, each at a concentration of 1000 mg/L, and stored at −20 °C in the refrigerator. Prior to analysis with GC-FPD and GC-ECD, working standards for organophosphate and synthetic pyrethroid insecticides were prepared by diluting the stock standards with ethyl acetate. The calibration curves of all pesticides range from 0.05–0.60 mg/kg.

The HBB sample was homogenized, weighed at 2.5 g into 50 mL centrifuge tubes, and spiked with 100 µL of working standards and internal standards. Ten milliliters of deionized water (DI) and 10 mL of acetonitrile (ACN) were added, and the sample was shaken for two minutes. The tubes were centrifuged at 3000 rpm for 5 min. The supernatant was transferred to a 15 mL centrifuge tube containing 4 g MgSO_4_, 1 g NaCl, and 1 g Na_3_C_6_H_5_O_7_ and shaken for 15 min. The slurry was then centrifuged at 3000 rpm for 5 min. The 5 mL of supernatant was transferred to a 15 mL centrifuge tube containing EMR lipid sorbent and immediately vortexed to disperse, then centrifuged at 3000 rpm for 5 min. The upper I layer was filtrated through a 0.22 µm syringe filter and transferred to sample vials to be ready for analysis of pesticide residue by GC-FPD and GC-ECD. The extract samples were analyzed using GC chromatography for pesticide residue organophosphate group separation by GC-FPD Model 7890B (columns used DB-1701, 0.25 mm, 30 m length, 0.25 μm film thickness, Agilent J & W column, Agilent Technologies, Santa Clara, CA, USA), pyrethroid group, and organochlorine using GC-ECD Model 7890B (column using HP5, 0.25 mm × ID × 30 m length × 0.25 μm film thickness, Agilent J & W column, Agilent Technologies, USA). The data were analyzed by OpenLab Chem Station software version C.01.07 (Agilent Technologies, Santa Clara, CA, USA).

#### 2.5.2. Organochlorine Pesticide Analysis (OCs)

The bee brood sample was extracted by the method described earlier [34]. The Ocs pesticides, i.e., beta-HCH, heptachlor, endosulfan, *o*,*p*′-DDT, *p*,*p*′-DDT, *o*,*p*′-DDE, *p*,*p*′-DDE, and *p*,*p*′-DDD, were analyzed. The 2.5 g of homogenized sample was extracted with 15 mL of acetonitrile in 1% acetic acid and shaken vigorously for 1 min. Then, 4 g of MgSO_4_, 1 g of NaCl, and 1.5 g of C_2_H_3_NaO_2_ were added and vortexed for 5 min. The solutions were centrifuged at 3000 rpm for 5 min, and then 5 mL of the extract was transferred into a 15 mL centrifuge tube containing EMR Lipid. The extracted sample was vortexed for 1 min and centrifuged at 3000 rpm for 5 min. The supernatant was pipetted into 2 mL of water to evaporate to dryness. The extracted sample was added to 1 mL of methanol and filtrated through a PTFE syringe filter prior to analysis by GC-ECD. The calibration curves range from 0.05–0.60 mg/kg.

#### 2.5.3. Polycyclic Aromatic Hydrocarbons (PAHs)

The powder-dried bee brood samples were analyzed for 16 PAHs using the modified method from a previous study [33]. The sample was injected into the GC-MS in spitless mode. The mass selective detector was operated in the electron impact (EI) mode set at 70 eV, and the mass scanning ranged in full scan mode from 50 to 350 *m*/*z*. Based on the selection mode (SIM) and the retention time of each PAH compared to the standard chromatogram, 16 PAHs were able to be identified. The 16-PAH mixed standard (Supelco EPA 610 PAH mix standard) included naphthalene (Nap), acenaphthylene (Acy), acenaphthene (Ace), fluorene (Flu), phenanthrene (Phe), anthracene (Ant), fluoranthene (Fla), pyrene (Pyr), benzo(a)anthracene (BaA), chrysene (Chr), benzo(b)fluoranthene (BbF), benzo(k)fluoranthene (BkF), benzo(a)pyrene (BaP), indenol (1,2,3-cd) pyrene (IcdP), dibenz(a,h)anthracene (DahA), and benzo(g,h,i)perylene (BghiP) in concentrations ranging from 1 to 50 μg/L. The first internal standard (Acy-D10) was used for Nap, Acy, Ace, Flu, Phe, Ant, Fla, and Pyr. The second internal standard (Per-D12) was used for BaA, Chr, BbF, BkF, BaP, IcdP, DahA, and BghiP. The concentration of individual detected PAHs was quantified by plotting their peak area ratio to the internal standard on the linear regression standard curve.

### 2.6. Statistical Analysis

Each experiment was performed three times, and the results were expressed as mean ± standard deviation. To examine the differences, one-way paired-samples *t*-test and one-way ANOVA, followed by DMRT (*p* < 0.05), were performed using IBM SPSS Statistics version 29.0.1.0.

## 3. Results

### 3.1. Bioactive Compounds

#### 3.1.1. Antioxidant Activity

Table 2 presents the antioxidant activities of HBB samples obtained from different farms situated in Northern Thailand. The findings indicated that all the samples examined exhibited antioxidant activity, with a significant degree of variability. The antioxidant capacity of ethanol-extracted samples was demonstrated by IC_50_ of DPPH (2.04–3.37) mg/mL, ABTS (21.22–33.91) mg/mL, and FRAP (50.07–104.15) mg AAE/100 g dry weight. The water-extracted samples demonstrated an IC_50_ of DPPH (10.67–84.97) mg/mL, ABTS (9.25–13.54) mg/mL, and FRAP (57.66–177.32) mg AAE/100 g dry weight. Except for ABTS HBB, samples extracted with water exhibited superior antioxidant properties, possibly attributed to the polar compounds extracted in an aqueous solution.

#### 3.1.2. Total Phenolic Content (TPC) and Total Flavonoids Content (TFC)

The Total Phenolic Content (TPC) and Total Flavonoid Content (TFC) of the HBBs are depicted in Table 3. The TPC and TFC of 70% ethanol extracted were within the range of 488.95–508.87 mg GAE/100 g dry weight and 4.71–12.98 mg QE/g dry weight, respectively. The TPC and TFC of DI water extracted range from 496.06–518.74 mg GAE/100 g dry weight and 5.32–21.60 mg QE/g dry weight, respectively. In both Total Phenolic Content (TPC) and Total Flavonoid Content (TFC) estimations, the water-extracted samples consistently displayed higher values, aligning with the increased antioxidant levels observed in these specific samples.

### 3.2. Phytochemical Analysis

This investigation confirmed the presence of phytoconstituents in the HBB extract, considering the molecular formula observed in *m*/*z* values, exact mass, and retention time. A detailed account of each annotated compound is presented in Table 4. Thirteen phenolic and flavonoid compounds, including 5Z-caffeoylquinic acid, gallic acid, protocatechuic acid-3-glucoside, protocatechuic acid, ellagic acid, rutin, orientin, epicatechin, vitexin, quercetin, pinobanksin, kaempferol, and caffeic acid, were identified. Five compounds, including protocatechuic acid, orientin, pinobanksin, kaempferol, and caffeic acid, were found in all the samples. Notably, all detected compounds exhibited a matching score, which is the value representing the match quality between the observed mass spectrum and the reference spectrum in a database exceeding 90%. 

### 3.3. Minerals

Table 5 provides a detailed account of the minerals of nutritional importance and content in the HBBs from different locations. Notably, the minerals K, Ca, Mg, and Na had the most prominent mineral content. The mineral concentrations identified in bee progeny samples are as follows: K (522.19–680.15 mg/100 g); Mg (58.23–64.29 mg/100 g); Ca (36.36–680.15 mg/100 g); and Na (27.11–43.35 mg/100 g), and among microelements, it was as follows: Zn (5.32–6.24 mg/100 g); Fe (3.99–5.25 mg/100 g); Cu (1.06–1.26 mg/100 g); and Mn (0.28–0.52 mg/100 g).

On the other hand, heavy metals, including Al, As, B, Ba, Ti, Cr, Cu, Mo, Ni, Pb, Sb, and Se, were detected, but their concentration was relatively low, as represented in Table 6.

### 3.4. Chemical Pollutants Analysis

The findings indicated the absence of any traces of harmful chemicals in all samples, even when considering the relatively high limit of detection (LOD) for the analysis OPs, 0.001–0.008 mg/kg; PYs, 0.001–0.007 mg/kg; OC, 0.002–0.007 and PAHs, 1.00–51.78 mg/kg. The linearity of the calibration curve was evaluated by investigating the correlation coefficient (R^2^) of the calibration curves. The *r* values were higher than 0.998 for all the analytes, as shown in Table 7. None of the polluting chemicals were detected in the bee brood samples.

## 4. Discussion

### 4.1. Antioxidant Activities of the Bee Brood

The antioxidant potential of HBBs suggests that antioxidants play a crucial role in reducing the production of harmful free radicals during food digestion, thus diminishing the risk of disease [35]. The DPPH and FRAP assays revealed higher values for extracts obtained with DI water compared to those extracted with 70% EtOH, whereas the ABTS radical-scavenging assay demonstrated greater efficacy for extracts obtained with 70% EtOH compared to those with DI water. The brood exhibits strong radical-scavenging activity in DPPH and ABTS tests, indicating its effectiveness in removing free radicals, as supported by prior research [35,36]. This trend is also evident in TPC and TFC, showing comparable levels in DI water and 70% EtOH extracts. According to the results obtained by Sawicki et al. [37], the TPC and TFC content in HBB showed high levels when compared with bee pollen, bee bread, and beeswax. Additionally, Lin et al. [6] reported that bee broods, especially drone broods, are rich in antioxidants such as ascorbic acid, coenzyme Q10, and all-trans-retinol. Phenolic and flavonoid compounds, known for their antioxidant properties, play a vital role. Some flavonoids exhibit metal-chelating properties crucial for inhibiting reactive oxygen species (ROS) formation and inhibiting enzymes like protein kinase and xanthine oxidase [37]. Moreover, flavonoids serve various functions in plants, including photoreception, antimicrobial activity, visual attraction, and herbivore repellency [38]. Carbohydrates with unbound aldehyde or ketone groups can function as reducing agents [39]. Excessive sugar intake is linked to unhealthy weight gain and diabetes onset, emphasizing the importance of carbohydrate restriction for maintaining a healthy weight [40,41]. The use of HBBs should not pose an elevated risk of obesity and associated complications for both children and adults [42].

### 4.2. Phytochemicals

Insects are gaining recognition as promising sources of health-boosting bioactive compounds, positioning them as potential functional foods [43]. Edible insects, such as silkworms, grasshoppers, mealworms, giant worms, and HBBs, are known for their antioxidant properties and potential role in preventing oxidative stress [44]. Torres-Castillo Certain et al. [45] found that edible insects contained bioactive compounds like β-carotene as well as flavonoids like kaempferol and quercetin. These bioactive substances are metabolized or absorbed by insects from the host plants that produce them as secondary metabolites, according to the results of Hussain et al. [44]. Phenolics, recognized as the most potent antiradical-containing compounds, activate enzymes that safeguard cells against damage related to oxidative stress [46]. Through UHPLC-QTOF-MS analysis, phytochemical compounds were identified in the HBB ethanolic extract, including gallic acid, caffeic acid, protocatechuic acids, kaempferol, quercetin, and rutin. Notably, additional compounds, such as caffeine, kaempferol, pi-nobanksin, quercetin, orientin, vitexin, rutin, caffeoylquinic acid, and proto-catechuic acid, were detected, aligning with previous studies by Sawicki et al. and Silva et al. [37,47]. This underscores the diverse array of beneficial compounds present in HBB, further highlighting its potential as a valuable source of bioactive components.

### 4.3. Mineral Content

The nutritional composition of HBB exhibits variation across different developmental stages yet consistently maintains high levels of protein, fat, carbohydrates, and minerals [37]. These minerals, linked to diverse health benefits such as bone health and optimal metabolic function in the human body, are noteworthy [47]. The brood displays elevated concentrations of potassium, with a comparatively lower concentration of sodium. High potassium and low salt content could have positive nutritional effects, especially for the segment of the population that suffers from hypertension; our results agree with those of Solayman et al. [48]. Regarding trace elements, this study indicates that the HBB possesses significant quantities of iron, zinc, and copper while revealing a lower amount of manganese, and the findings are in agreement with previous studies [1,4,5,6,8,9,10]. Calcium is crucial for bone and dental health, heart rhythm, muscle function, nerve activity, hormone regulation, and blood pressure [49]. Adequate daily calcium intake is crucial for overall well-being and can be achieved through a diverse diet incorporating calcium-rich foods [50]. Sufficient dietary calcium is particularly important for supporting bone growth, with pregnant women requiring it for fetal bone development and postmenopausal women prioritizing calcium intake to reduce the risk of osteoporosis [34].

Iron deficiency, the primary cause of anemia globally, arises from reduced iron intake, blood loss, or impaired iron absorption. This deficiency, known as iron deficiency anemia (IDA), is a significant global health issue affecting millions of people, especially women and children, in developing and underdeveloped regions [25].

Zinc is essential for various metabolic processes, including protein synthesis, transcription, and DNA replication [51]. Zinc deficiency affects human health and can manifest in symptoms like stunted growth, appetite loss, and impaired smell and taste [44,52]. Severe zinc deficiency can impact various organ systems, including the immune, gastrointestinal, central nervous, skeletal, and reproductive systems [53]. Assuming favorable bioavailability, the inclusion of HBB in one’s diet could be anticipated to provide essential minerals of nutritional significance at noteworthy levels. Contrastingly, the current study indicates that HBB samples from various apiaries do not contain any heavy metals exceeding safe levels.

### 4.4. Food Safety

Pesticide contamination in honey bee larvae poses a significant concern, as it has the potential to adversely impact bee health, leading to increased mortality [54]. Medina and Ravoet et al. and Ravoet et al. [55,56] found pesticide residues commonly found in wax combs, often characterized by their lipophilic nature or dissolved in oil-based carriers. These substances may be introduced into the hive through contaminated nectar and pollen by honey bees or by beekeepers seeking to control parasites, according to the results of Karazafiris [57]. This contamination can result in the transfer of pesticides into various food resources, including honey, beebread, and bee broods [57,58]. Beeswax, utilized in the food, cosmetics, and pharmaceutical industries, may expose humans to pesticides [51]. By identifying environmental chemicals, such as pesticides and polycyclic aromatic hydrocarbons (PAHs), as potential contaminants, as reported by Arjin et al. and Amekawa et al. [59,60], this study did not determine if the samples surpassed the limit of detection (LOD) for hazardous compounds, indicating the absence of such substances in the examined HBB samples. It is imperative to establish chemical safety for bee broods to gain consumer acceptance, as the presence of harmful elements, pesticide residues, or other environmental contaminants can diminish their nutritional value and nutritional benefits [61].

## 5. Conclusions

This study found that HBB was a promising and sustainable food source, given its antioxidant properties, an abundance of phenolic compounds, and rich mineral content, potentially offering various health benefits. Hydrophilic antioxidants play a substantial role in antioxidant activity levels, which can vary significantly depending on the bee’s diet, the time of year, and the specific environmental conditions, so the limitations of this study did not account for the potential seasonal variations that might affect the composition of HBB, including variances in the levels of minerals, such as sodium, potassium, etc., in the HBB, which are probably influenced by their environments, ecological traits, and the diverse plant sources they feed on. The current research indicates that HBBs from different apiaries are free of harmful chemicals and contaminants, confirming their safety for consumption. Embracing HBB as a food source contributes to food and nutrition security by presenting a locally available, natural, and sustainable option that complements diets and enhances nutritional value. Future studies should include samples collected across different seasons to understand how seasonal changes impact the nutritional and chemical characteristics of HBB and how awareness of HBB consumption has the potential to yield health benefits.

## Figures and Tables

**Table 1:** Geographical locations of bee brood samples collection and their water content (%).

Sample	Apiary Location	Water Content (%)
HB-NT-1	Nong Muang Kai District, Phrae Province	73.95
HB-NT-2	Song District, Phrae Province	77.51
HB-NT-3	San Sai District, Chiang Mai Province	76.61
HB-NT-4	Sung Men District, Phrae Province	71.59
HB-NT-5		68.75

**Table 2:** Antioxidant activity (DPPH, ABTS, and FRAP) in HBB extracted samples of different honey bee farm locations.

ID Farms	DPPH-Scavenging Activity			ABTS Radical-Scavenging Activity			Ferric Ion Reducing Antioxidant Power		
	IC_50_ (mg/mL)			IC_50_ (mg/mL)			(mg AAE/100 g Dry Weight)		
Extracted	70% EtOH	DI Water	*p*-Value	70% EtOH	DI Water	*p*-Value	70% EtOH	DI Water	*p*-Value
HB-NT-1	3.20 ± 0.34 ^a^	39.68 ± 6.72 ^b^	0.007 **	27.91 ± 6.47 ^ab^	13.37 ± 1.87 ^a^	0.063	94.90 ± 4.85 ^a^	146.92 ± 13.17 ^b^	0.038 **
HB-NT-2	2.04 ± 0.10 ^b^	10.67 ± 2.64 ^c^	0.032 **	33.72 ± 1.69 ^a^	9.25 ± 1.21 ^b^	0.003 **	104.15 ± 25.43 ^a^	145.15 ± 6.67 ^b^	0.122
HB-NT-3	2.43 ± 0.17 ^b^	14.39 ± 4.54 ^c^	0.004 **	33.90 ± 3.24 ^a^	11.33 ± 1.7 ^ab^	0.015 **	98.48 ± 40.16 ^a^	177.32 ± 14.53 ^a^	0.037 **
HB-NT-4	3.37 ± 0.37 ^a^	84.97 ± 14.02 ^a^	0.010 **	33.91 ± 9.48 ^a^	13.54 ± 2.86 ^a^	0.077	79.21 ± 14.94 ^ab^	119.94 ± 12.43 ^c^	0.105
HB-NT-5	3.28 ± 0.07 ^a^	77.52 ± 10.3 ^a^	0.000 **	21.22 ± 2.92 ^b^	9.40 ± 0.96 ^b^	0.025 **	50.07 ± 4.11 ^b^	57.66 ± 5.42 ^d^	0.011 **

DPPH: 2,2-diphenyl-1-picrylhydrazyl; ABTS: 2,2-casino-bis (3-ethylbenzothiazoline-6-sulfonic acid). FRAP: ferric ion reducing antioxidant power; EtOH: ethanol; DI water: deionized water. The results are presented as mean values ± standard deviation. Different superscript letters within the same column indicate a significant difference in antioxidant activity between locations (*p* ≤ 0.05). ** Significant differences for data analyzed using one-way paired-samples *t*-test (*p* < 0.05).

**Table 3:** Total phenolic content (TPC) and total flavonoid content (TFC) in HBB-extracted samples of different honey bee farm locations.

ID Farms	Total Phenolic Content			Total Flavonoid Content		
	(mg GAE/100 g Dry Weight)			(mg QE/g Dry Weight)		
Extracted	70% EtOH	DI Water	*p*-Value	70% EtOH	DI Water	*p*-Value
HB-NT-1	488.95 ± 14.28	496.06 ± 10.93 ^b^	0.421	12.53 ±3.80 ^b^	4.32 ± 3.25 ^b^	0.074
HB-NT-2	501.37 ± 18.01	508.80 ± 6.45 ^ab^	0.422	5.34 ± 1.71 ^c^	9.15 ± 4.48 ^ab^	0.325
HB-NT-3	497.93 ± 15.87	518.74 ± 14.92 ^ab^	0.001 **	4.46 ± 3.07 ^c^	6.14 ± 5.04 ^b^	0.321
HB-NT-4	508.87 ± 20.16	508.19 ± 13.14 ^ab^	0.961	18.88 ± 3.59 ^a^	21.60 ± 9.81 ^a^	0.758
HB-NT-5	491.62 ± 4.29	512.60 ± 16.10 ^a^	0.040 **	4.98 ± 3.25 ^c^	21.48 ± 7.98 ^a^	0.097

GAE: gallic acid equivalents; QE: quercetin equivalents. The results are presented as mean values ± standard deviation. Different superscript letters within the same column indicate a significant difference in total phenolic and total flavonoid content between locations (*p* ≤ 0.05). ** Significant differences for data analyzed using one-way paired-samples *t*-test (*p* < 0.05).

**Table 4:** Phytochemical identification of different honey bee farm locations by LC-Q-TOF/MS.

No.	Phytochemicals	Mass	RT	Score	Formula	*m*/*z*	Location of Samples				
							HB-NT-1	HB-NT-2	HB-NT-3	HB-NT-4	HB-NT-5
1	5Z-Caffeoylquinic acid	354.097	1.813	90.34	C_16_ H_18_ O_9_	353.089	+	−	+	−	+
2	Gallic acid	170.019	2.166	90.55	C_7_ H_6_ O_5_	188.054	+	+	−	+	−
3	Protocatechuic acid-3-glucoside	316.078	3.45	89.35	C_13_ H_16_ O_9_	334.111	+	+	−	−	−
4	Protocatechuic acid	154.026	4.381	85.99	C_7_ H_6_ O_4_	153.019	+	+	+	+	+
5	Ellagic acid	302.007	9.095	88.52	C_14_ H_6_ O_8_	303.014	+	−	+	−	−
6	Rutin	610.152	9.786	97.83	C_27_ H_30_ O_16_	633.141	+	+	+	+	-
7	Orientin	448.1	10.988	98.76	C_21_ H_20_ O_11_	447.092	+	+	+	+	+
8	Epicatechin	290.078	11.372	99.57	C_15_ H_14_ O_6_	289.071	+	+	−	−	−
9	Vitexin	432.104	13.168	93.96	C_21_ H_20_ O_10_	477.103	−	−	+	+	+
10	Quercetin	302.042	15.235	98.91	C_15_ H_10_ O_7_	301.035	+	+	+	+	−
11	Pinobanksin	272.068	15.586	99.28	C_15_ H_12_ O_5_	273.075	+	+	+	+	+
12	Kaempferol	286.047	17.77	99.29	C_15_ H_10_ O_6_	287.054	+	+	+	+	+
13	Caffeic acid	180.042	29.383	98.72	C_9_ H_8_ O_4_	203.031	+	+	+	+	+

RT is short for “retention time” here (+) = presence of constituents; (−) = absence of constituents.

**Table 5:** Mineral content (mg/100 g dry matter basis) in the HBB samples from different honey bee farm locations.

Minerals	HB-NT-1	HB-NT-2	HB-NT-3	HB-NT-4	HB-NT-5
Ca	69.60 ± 1.22 ^a^	43.68 ± 6.70 ^b^	67.87 ± 2.44 ^a^	66.69 ± 10.54 ^a^	36.36 ± 2.10 ^b^
Co	ND	ND	ND	ND	ND
K	579.81 ± 21.28 ^b^	533.36 ± 3.17 ^c^	680.15 ± 3.36 ^a^	530.59 ± 10.31 ^c^	522.19 ± 9.02 ^c^
Mg	62.28 ± 1.07 ^a^	62.97 ± 0.84 ^a^	64.29 ± 0.41 ^a^	58.44 ± 2.28 ^b^	58.23 ± 0.47 ^b^
Na	36.53 ± 0.07 ^c^	40.97 ± 0.96 ^b^	27.11 ± 0.04 ^e^	30.41 ± 1.30 ^d^	43.35 ± 0.85 ^a^
Zn	6.24 ± 0.19 ^a^	6.11 ± 0.17 ^a^	5.78 ± 0.06 ^b^	5.59 ± 0.26 ^bc^	5.32 ± 0.13 ^c^
Fe	5.08 ± 0.63 ^a^	5.25 ± 0.30 ^a^	4.05 ± 0.03 ^b^	3.99 ± 0.07 ^b^	4.02 ± 0.25 ^b^
Cu	1.26 ± 0.04 ^a^	1.06 ± 0.02 ^c^	1.15 ± 0.01 ^b^	1.16 ± 0.01 ^b^	1.11 ± 0.03 ^b^
Mn	0.51 ± 0.02 ^a^	0.44 ± 0.01 ^b^	0.28 ± 0.00 ^d^	0.52 ± 0.01 ^a^	0.35 ± 0.01 ^c^

The results are presented as mean values ± standard deviation. Different superscript letters within the same row indicate a significant difference in mineral content between locations (*p* ≤ 0.05). ND—Not detected.

**Table 6:** Heavy metals (mg/100 g dry matter basis) in the HBB samples from different honey bee farm locations.

Heavy Metal	HB-NT-1	HB-NT-2	HB-NT-3	HB-NT-4	HB-NT-5
Al	2.01 ± 0.20 ^a^	1.43 ± 0.23 ^ab^	1.55 ± 0.45 ^ab^	1.89 ± 0.42 ^a^	1.14 ± 0.12 ^b^
Ag	ND	ND	ND	ND	ND
As	0.04 ± 0.02	0.05 ± 0.30	0.02 ± 0.01	0.04 ± 0.02	0.04 ± 0.00
B	0.62 ± 0.02 ^a^	0.34 ± 0.04 ^b^	0.23 ± 0.04 ^b^	0.25 ± 0.04 ^b^	0.23 ± 0.02 ^b^
Ba	0.15 ± 0.01 ^a^	0.06 ± 0.03 ^b^	0.12 ± 0.00 ^a^	0.12 ± 0.04 ^a^	0.05 ± 0.01 ^b^
Be	00 ± 0.01	00 ± 0.00	00 ± 0.00	00 ± 0.00	00 ± 0.00
Cd	ND	ND	ND	ND	ND
Ti	0.05 ± 0.00	0.11 ± 0.05	0.06 ± 0.00	0.09 ± 0.06	0.07 ± 0.02
Tl	ND	ND	ND	ND	ND
Cr	0.08 ± 0.02 ^b^	0.13 ± 0.00 ^a^	0.09 ± 0.01 ^b^	0.02 ± 0.00 ^c^	0.04 ± 0.02 ^c^
Mo	0.03 ± 0.02 ^a^	0.02 ± 0.00 ^ab^	0.02 ± 0.00 ^ab^	0.01 ± 0.00 ^b^	0.01 ± 0.00 ^ab^
Ni	0.02 ± 0.03	ND	0.01 ± 0.018	ND	ND
Pb	0.02 ± 0.02	ND	ND	ND	0.02 ± 0.041
Sb	0.04 ± 0.03	0.03 ± 0.00	0.03 ±0.02	0.03 ± 0.02	0.03 ± 0.017
Se	0.03 ± 0.02 ^b^	0.02 ± 0.00 ^b^	0.07 ± 0.02 ^a^	0.04 ± 0.02 ^b^	ND
Si	6.12 ± 1.51 ^a^	4.49 ± 0.38 ^b^	4.26 ± 0.12 ^b^	4.66 ± 0.11 ^b^	3.37 ± 0.15 ^b^
V	0.00 ± 0.00 ^b^	0.00 ± 0.00 ^bc^	0.02 ± 0.00 ^a^	0.00 ± 0.00 ^bc^	0.00 ± 0.00 ^c^

The results are presented as mean values ± standard deviation. Different superscript letters within the same row indicate a significant difference in heavy metals between locations (*p* ≤ 0.05). ND—Not detected.

**Table 7:** Limit of detection, working standard range, and correlation coefficient of the calibration curve for the chemical pollutants analysis.

Parameter (Unit)	Chemical Pollutants			
	OPs	PYs	OC	PAHs
LOD (mg/kg^−1^)	0.001–0.008	0.001–0.007	0.002–0.007	1.00–51.78
LOQ (mg/kg^−1^)	0.020–0.060	0.010–0.020	0.010–0.030	11.00–71.00
Working standard range (μg/L)	50–600	50–600	50–600	1–50
Correlation of the calibration curve (R^2^)	0.9919–0.9993	0.99192–0.9992	0.9907–0.9988	0.9919–0.9998

## Data Availability

The original contributions presented in the study are included in the article, further inquiries can be directed to the corresponding authors.
